# Decompression Illness After Technical Diving Session in Mediterranean Sea: Oxidative Stress, Inflammation, and HBO Therapy

**DOI:** 10.3390/ijms252111367

**Published:** 2024-10-22

**Authors:** Simona Mrakic-Sposta, Andrea Brizzolari, Alessandra Vezzoli, Carmela Graci, Attilio Cimmino, Tommaso Antonio Giacon, Cinzia Dellanoce, Alessandra Barassi, Giovanni Sesana, Gerardo Bosco

**Affiliations:** 1 Institute of Clinical Physiology, National Research Council (IFC-CNR), Piazza dell’Ospedale Maggiore, 3, 20142 Milan, Italy; alessandra.vezzoli@cnr.it (A.V.); cinzia.dellanoce@ifc.cnr.it (C.D.); 2 ASST Grande Ospedale Metropolitano Niguarda, Piazza dell’Ospedale Maggiore, 3, 20142 Milan, Italy; graci.carmela@gmail.com (C.G.); attiliocimmino@ospedaleniguarda.it (A.C.); giovanni.sesana@ospedaleniguarda.it (G.S.); 3 Department of Biomedical Sciences, University of Padova, 35131 Padova, Italy; andrea.brizzolari@unipd.it (A.B.); tommasoantonio.giacon@studenti.unipd.it (T.A.G.); 4 Department of Health Sciences, Università degli Studi of Milan, 20142 Milan, Italy; alessandra.barassi@unimi.it

**Keywords:** SCUBA diving, oxidative stress, inflammation, decompression sickness, HBO therapy, arterial gas embolism, nitric oxide

## Abstract

SCUBA diving poses risks due to pressure changes during descent (compression) and ascent (decompression). Decompression sickness (DCS) occurs due to gas bubble formation as the pressure decreases, causing joint pain, numbness, dizziness, or even paralysis and death. Immediate treatment involves 100% oxygen to help eliminate inert gases and hyperbaric oxygen therapy (HBOT), which is essential to reduce gas emboli formation and inflammation, thus improving symptoms. We evaluated oxy-inflammation biomarkers in the saliva and urine of nine subjects pre- and post-technical dive on the Haven wreck (GE, Italy). A case of DCS occurred during the dive. The injured diver was treated immediately with O_2_ and transported to the hyperbaric center of “ASST Ospedale Ca Granda” in Milan. He was treated following the U.S. Navy Treatment Table 5 at 2.8 ATA and the day after with Table 15 at 2.4 ATA. Venous blood and urine samples were collected before and after each HBO treatment. Our study shows that dive increased oxy-inflammation biomarkers (ROS +126%; lipid peroxidation +23%; interleukins-6 +81%, -1β +19%, and TNFα +84%) and nitric oxide metabolites levels (+36%). HBOT after a DCS episode reduced oxidative stress, lowering the very high marker of lipid peroxidation (8-iso-PGF2α), and inhibited inflammatory interleukins. Overall, HBOT improved physiological responses in the diver affected by DCS.

## 1. Introduction

Self-contained underwater breathing apparatus (SCUBA) diving is associated with risks related to the increase in pressure (compression) at the dive’s beginning and its decrease (decompression) at the end. The term decompression illness (DCI) describes two separate mechanisms that result in overlapping symptoms: arterial gas embolism (AGE) and decompression sickness (DCS) [1]. DCS occurs with the formation and increase in the size of bubbles when the sum of total gas tension exceeds the local pressure. This can be caused by the gas trapped as a result of airway obstruction or by the presence of pulmonary blebs [2]. In a diving context, this state is caused by the increase in partial pressure of inert gasses that occurs when the gas (N_2_ or He) is breathed at high pressure [2]. DCS can cause several symptoms (including joint pain, numbness, paresthesia, dizziness/vertigo, lymphatic disorders, fatigue, abdominal pain, and paralysis) [3,4]. DCS cases are typically divided into Type I (entailing mostly cases with pain symptoms) or Type II (includes neurological and cardiopulmonary cases) [3]. The first on-site treatment of DCI is to supply 100% O_2_. Pure O_2_ washes inert gas from the lungs and establishes the highest possible inert gas gradient from tissue to alveoli. In this way, perfusion itself removes inert gas from tissues to lungs and from bubbles to tissues by diffusion [5], mitigating tissue hypoxia caused by bubble-induced ischemia, mechanical injuries, and biochemical damage [6]. Divers with suspected symptoms of DCI have to be referred to the closest hyperbaric center available for an emergency treatment with hyperbaric oxygen therapy (HBOT) [7,8]. Based on U.S. Navy treatment tables [9], HBOT is administered following pre-established compression profiles that already proved to be safe and effective in reducing the symptoms of DCI through the interruption of the pathophysiological process of ischemia, inflammation, and endothelial damage.

Diving-related physical effort and exposure to high oxygen partial pressure (pO2) can raise the production of reactive oxygen species (ROS), oxidative stress biomarkers including 8-isoprostane (8-iso-PGF2α), products of lipid peroxidation [10], and 8-oxo-7,8-dihydro-deoxyguanosine (8-OH-dG), the index of DNA damage [11], investigated in both warm [12] and cold water [13]. Despite the activation of antioxidant defenses, the hyperbaric environment may increase ROS and reactive nitrogen species (RNS) generation [14,15], overwhelming endogenous antioxidant capacity [16] and resulting in an oxidative stress condition. Furthermore, an excess of ROS can promote the release of pro-inflammatory cytokines including interleukin beta (IL-1β) [17], interleukin-6 (IL-6) [18], and tumor necrosis factor-α (TNFα) [19], involved in mediating neutrophil action at the level of the lungs [20]. On the other hand, IL-6 release seems to play a key role in the production of energy to sustain the physical effort that occurs during the dive by stimulating glucose production and activating lipid metabolism through the stimulation of fatty acid catabolism [21]. Indeed, the diving-related environmental conditions increase the circulating level of creatine kinase (CK) and lactate dehydrogenase (LDH) after surfacing as a consequence of myocyte activation and the alteration of myocyte membrane integrity [22]. CK measured in the blood is a marker of muscle damage.

Adaptation to a hyperbaric environment implies an increase in nitric oxide (NO) production, as observed by several authors who measured nitrates and nitrites (NOx), NO derivatives, during the deep phase of the dive [23]. NO plays a crucial role in the regulation of blood vessel diameter and flow, as well as being a signaling molecule for regulating vascular tone [24].

Dehydration has been proposed as a DCS risk: the increasing blood viscosity and the altering microcirculatory perfusion may promote bubble formation [25]. It seems dehydration could also alter inert gas clearance by reducing the blood flow to poorly perfused tissues, or it may decrease surface tension and thereby facilitate bubble formation [26].

Within an experimental session aimed at the analysis of oxy-inflammation after a dive, we reported a case of DCS in an expert technical SCUBA diver. Thereafter, the aim of this study turned to the analysis of oxy-inflammation during the treatment of DCS.

The examined case demonstrates the importance of (a) the prompt diagnosis of decompression illness, (b) timely oxygen administration, and (c) adequate HBOT after DCS. In addition, the novelty of this evaluation consists of assessing the kinetics of biomarker levels both pre-dive and throughout the DCS evolution.

## 2. Results

**Figure 1:**
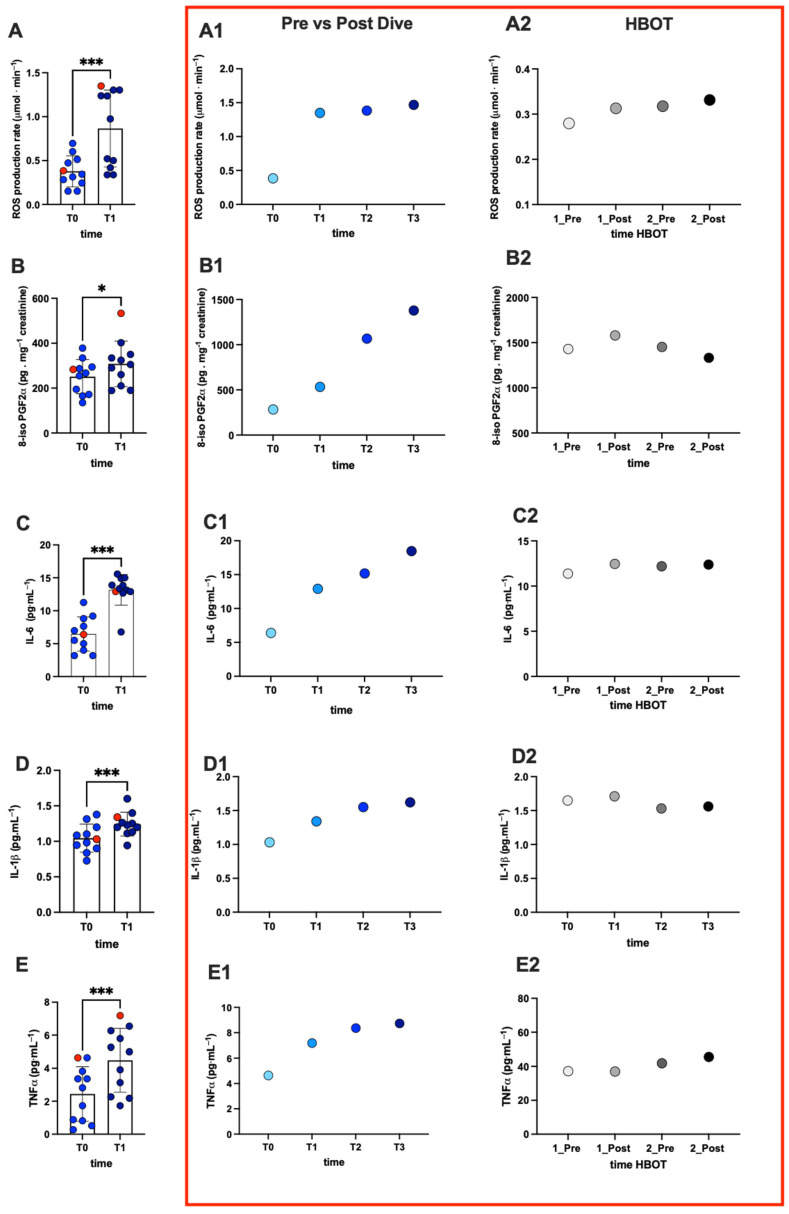
Panel plots of the oxy-inflammation biomarkers in divers and the evolution of concentration in the DCS-affected subject. Histogram plot of (**A**) salivary ROS production rate, (**B**) urinary lipid peroxidation (8-isoPGF2α), and salivary (**C**) IL-6, (**D**) IL-1β, and (**E**) TNF-α levels in all divers pre- and post-immersion. The red dot indicates the subject of the DCS case. Statistically significant differences comparisons are displayed as follows: *, *p* < 0.05; and ***, *p* < 0.001. At the right panel, inside the red box, the time course of (**A1**,**A2**) ROS production rate, (**B1**,**B2**) lipid peroxidation, (**C1**,**C2**) IL-6, (**D1**,**D2**) IL-1β, and (**E1**,**E2**) TNF-α levels from T0 to T3 in saliva (T0: 30 min before the dive; T1: 30 min after surfacing and DCS symptom onset; T2: 3 h after DCS symptom onset; T3: 6 h after DCS symptom onset) and plasmatic values for the two HBOT sessions in the injured subject.

The oxy-inflammation levels at pre- (T0) vs. post-dive (T1) in saliva and/or urine in all divers and, in the red-bordered graphics, the kinetics of the subject with DCS are displayed in Figure 1. Overall, a significant increase after immersion was recorded in ROS production (A, +126%), lipid peroxidation (B: +23%), and inflammatory markers (C, IL-6: +81%; D, IL-1β +19%; and E, TNF-α: +84%).

In the DCS-affected subject, the ROS level increased at post-dive (T1): (A1, +255%), lipid peroxidation (B1: +88%), inflammatory markers (C1, IL-6: +103%; D1, IL-1β: +34%; and E1, TNF-α: +54%). At T2 and T3, the data showed a continuous change/increase in values as the hours passed, highlighting an oxy-inflammation state in the acute phase. Figure 1 also shows the oxy-inflammation levels during HBOT in the DCS case. The effects of HBOT in plasma on oxy-inflammation levels before (1_Pre) and after (1_Post) the first HBOT session and before (2_Pre) and after (2_Post) the second HBOT session of treatments are reported.

The data show an increase in ROS production (A2: +12%) in post-HBOT sessions, as reported in the literature [27,28]. Furthermore, post-HBOT during the first session, an increase in lipid peroxidation (B2: +10%) and inflammatory markers (C2, IL-6: +9%; D2, IL-1β; +3.5%, and TNF-α: +0.5%) was recorded. On the second day of treatment, this trend tends to decrease.

**Figure 2:**
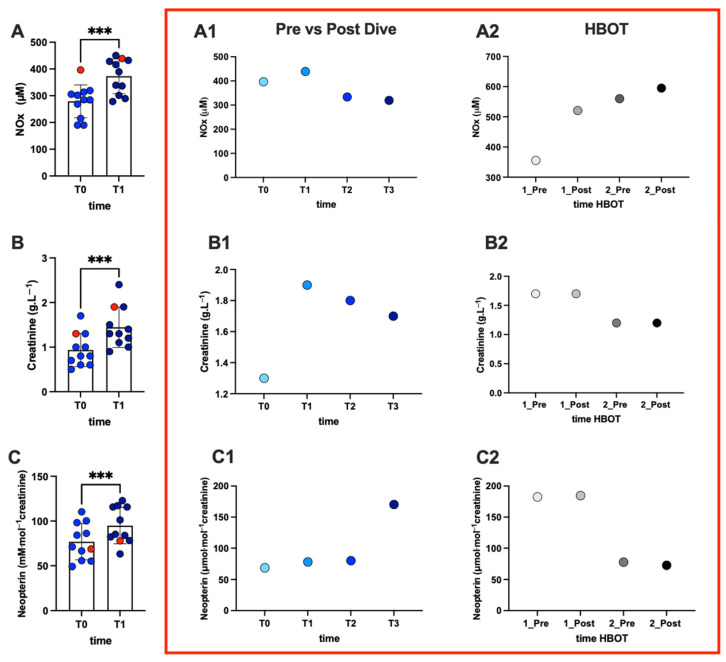
Panel plots of NO metabolites and renal status in divers and the evolution of concentration in the DCS-affected subject. Histogram plot of urinary (**A**) NO metabolites, (**B**) creatinine, and (**C**) neopterin concentration in all divers pre- and post-immersion. The red dot indicates the subject of the DCS case. Statistically significant differences comparisons are displayed as follows: ***, *p* < 0. 001. At the right panel, inside the red box, the time course of (**A1**,**A2**) NOx, (**B1**,**B2**) creatinine, and (**C1**,**C2**) neopterin from T0 to T3 (T0: 30 min before the dive; T1: 30 min after surfacing and DCS symptom onset; T2: 3 h after DCS symptom onset; T3: 6 h after DCS symptom onset), and in the two HBOT sessions in the injured subject.

Overall, a significant increase after immersion was recorded in NO metabolite levels (Figure 2; A, NOx + 34%). Also, in the injured subject, NOx increased (A1, NOx + 11%). Post-HBOT during the first session, NOx levels increased (A2, NOx: +46%). On the second day of treatment, the trend of NOx continued increasing.

Renal biomarker status levels at pre- (T0) vs. post-dive (T1) in all divers and, in the red-bordered graphics, the kinetics of the subject with DCS are shown in Figure 2. Overall, a significant increase after immersion in creatinine (B: +55%) and neopterin concentration (C: +23%) were measured. In the injured subject, there was an increase in the post-dive (T1) levels of creatinine (B1: +46%) and neopterin concentration (C: +13%). In the 1st session of HBOT, no changes in creatinine and neopterin levels were observed, and in the 2nd session of HBOT treatment, the trend tends to decrease in neopterin levels +5%.

Table 1 reports the values of uric acid, urea, Na, K, Cl, Mg, Ca, and total protein in urine measured at pre- and post-dive in all subjects. Furthermore, Table 1 shows the reported data recorded by the injured subject at baseline, post-immersion (T1), 3 h (T2), 6 h after DCS symptoms (T3), and pre/post-HBOT on the first (T1_Pre/T1_Post) and second day (T2_Pre/T2_Post) of treatment.

### 2.1. Cardio-Muscular Biomarkers

We observed that cTnI remained unchanged before and after any hyperbaric treatment, and the same was true for CK-MBm (1_pre: 0.56 ng/mL vs. 1_post: 0.53 ng/mL; 2_pre: 0.53 ng/mL vs. 2_post: 0.56 ng/mL). CK levels were unchanged before and after the first hyperbaric treatment (1_pre: 89 U/L vs. 1_post: 91 U/L), while they increased after the second treatment (2_pre: 51 U/L vs. 1_post: 76 U/L).

### 2.2. Scale Physical Fatigue, Pain, and VAS

**Figure 3:**
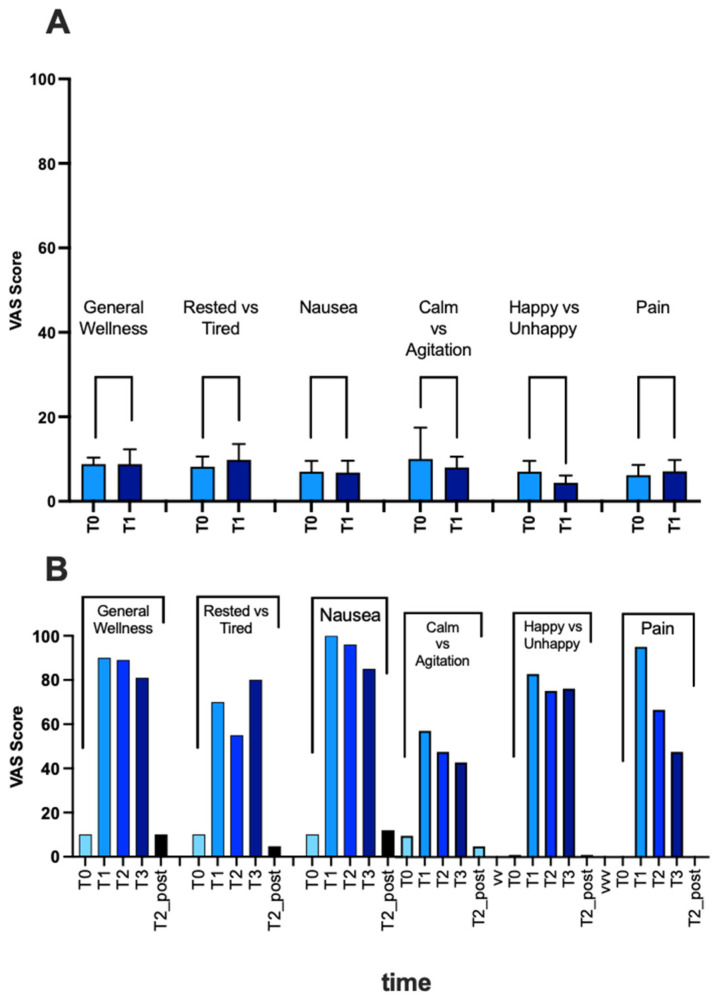
In the histograms, the VAS scores for general wellness, tiredness, nausea, agitation, happiness, and pain are recorded (**A**) for SCUBA divers all together; (**B**) from T0 to T3 (T0: 30 min before the dive; T1: 30 min after surfacing and DCS symptom onset; for T2: 3 h after DCS symptom onset; T3: 6 h after DCS symptom onset) and after the last HBOT treatment (T2_post) in the injured subject.

The BORG scale score for the diving group was 12.09 ± 1.04, while that of the injured subject was 13. The histogram in Figure 3A (VAS) reports the subject mood scores in SCUBA, excluding the injury subject. The diving group did not report any general malaise, unhappiness, or pain, and the changes were due to the elevated values of the injured subject. In fact, immediately after the immersion, concomitantly to DCS, there is an increase in all items of general malaise (tiredness, nausea), unhappiness, and pain, which lasted until arrival at the hospital but resolved after two HBOT treatments (Figure 3B).

## 3. Discussion

We reported the case of a DCS episode that occurred in an expert diver immediately after a technical dive. The strength of this study is that we have been able to analyze and compare both the DCS-affected diver and the other divers in the same group, therefore highlighting the oxy-inflammation in a subject that developed DCS compared with others that did not, and also isolating the effects of HBOT as a treatment. The Borg scale value was 15 (hard), probably due to the technical dive-related stressors, including equipment weight, breathing resistance, high pO_2_ and pN_2_ values, and decompression procedures.

It has been hypothesized that oxidative stress in underwater breathing apparatus (SCUBA) divers is caused by exposure to an increased pO_2_, resulting in a hyperoxic condition [29]. Diving-related physical effort, an increase in breathing resistance, and coldness may increase free radical production [30]. Furthermore, technical divers must breathe enriched air nitrox (EAN) mixtures, consisting of a lower N_2_ percentage and a higher O_2_ percentage, with respect to air, during the decompression stops to create a favorable N_2_ gradient from the tissues to the alveoli, to reduce the formation of venous gas emboli (VGE) and therefore reduce the risk of DCS onset [31,32]. Our data show that increased levels of hyperoxia-induced ROS readily react with lipids, proteins, and nucleic acids, leading to oxidative stress [10] and the activation of nuclear factor-kappa B (NFκB) and nuclear factor (erythroid-derived)-like 2 (NRF2) [33]. An ROS increase was also observed in the injured diver. Oxidative stress plays a crucial role in the mechanism of circulating microparticles (MPs) being released during diving [34,35], leading to neutrophil activation [36] and perivascular adherence [37] associated with DCS. Similarly to ROS, we found an increase in the 8-iso-PGF2α value, the index of lipid peroxidation, which occurred as a consequence of diving, which is associated with structure disturbance and the function loss of biological membranes induced by oxidative stress [38]. 8-iso-PGF2α was particularly elevated in the injured subject. During HBOT, human body can activate several mechanisms to counterbalance ROS generation including vasoconstriction [39] and mobilization of endogenous antioxidant defenses [40]. Furthermore, the 8-iso-PGF2α value increased only slightly after HBOT, and the resulting absolute values were similar to those recorded at the pre-dive observation (T0). This is probably due to the HBOT-related protective role against oxidative stress [40]. ROS concentrations slightly above the baseline value can explain the exerted beneficial effects of regulating cell signaling cascades on the inflammation status.

Regarding inflammation, we observed a significant augmentation of pro-inflammatory cytokines IL-6, IL-1β, and TNFα after surfacing. Increased levels of circulating ROS can activate (NF-kB) [41], which promotes the expression of several inflammation-related genes [42,43]. On the other hand, the augmentation of IL-6 may be also the consequence of physical effort [44] that leads to an acute phase response [45]. Furthermore, IL-1β elevation seems to be related to high partial pressures of breathed gases instead of a response to decompression [46]. In our DCS case, we found the rise in all pro-inflammatory biomarkers described above to be a consequence of ROS-related pro-inflammatory genes [42] that may be involved in the DCS process [47]. HBOT, the definitive DCS treatment [48], can inhibit pro-inflammatory interleukins including IL-6 and IL-1β [28,49], while seems to stimulate the release of anti-inflammatory IL-1α [50]. Furthermore, HBOT decreases levels of the pro-inflammatory cytokines interferon-γ (IFN-γ), NF-κB, and TNF-α [51]. In our case, we observed slightly raised pro-inflammatory biomarker levels as a consequence of the beneficial effect of HBOT.

Contrary to data observed previously, wherein NOx values returned close to pre-dive levels [23], we observed a NOx increase post-dive. An increase in NOx indicates an intense NO production level to regulate the vasoconstriction/vasodilation mechanism, probably to adapt the body to a hyperbaric environment [23,52]. Furthermore, increased NOx levels, products of NO oxidation [53], may indicate a consequence of increased O_2_ availability. This may be due to the physical effort that raises endothelial nitric oxide synthase (eNOS) gene expression in the lungs, increasing NO production [54]. Physical activity may promote endothelial function by increasing vascular endothelial growth factor-induced angiogenesis (VEGF), leading to elevated NO generation and thus increasing serum/plasma NO levels [55]. In our injured diver, we observed a decrease in NOx, probably due to a reduction in NO availability caused by free radicals [56]. HBOT can restore NO generation by stimulating NOS expression [57] and reducing oxidative stress [40].

Neopterin levels usually rise during systemic oxidative stress, as previously described [58]. Similarly to the biomarkers previously described, post-dive neopterin levels tend to increase, confirming what is already shown with respect to other inflammatory markers after surfacing [59].

As reported in Table 1, we found a significant reduction in uric acid after dive. Uric acid is an important component of endogenous antioxidant defenses [60]. Its reduction after a dive may be the consequence of the activation of endogenous antioxidant defenses against oxidative stress.

A decrease in urea values is likely to reduce dive-related water loss due to a water-conserving mechanism in a condition of dehydration [61]. Regarding electrolytes, except for magnesium (Mg), we did not find any other significant difference in the sodium (Na), potassium (K), chloride (Cl), calcium (Ca), and total protein levels. A Mg decrease may be related to two different mechanisms. First, Mg, in its ionic form Mg^2+^, reacts with ATP to form the Mg-ATP complex, the primary energy source to sustain physical effort [62]. Second, a Mg decrease may be related to inflammatory status [63] and oxidative stress onset [64].

In our DCS case, except for K after the dive (Table 1), the electrolytes decrease results from DCS-related dehydration and oxygen breathing after DCS onset. On the other hand, electrolytes may be reabsorbed to avoid an exercise-related electrolyte imbalance. Dehydration has been proposed as a DCS risk: the increasing blood viscosity and the altered microcirculatory perfusion may promote bubble formation [12]. A K increase after diving may be due to its role in nerve stimulation and muscle contraction during exercise. The increase in electrolytes after the first HBOT treatment might be the consequence of a soft dehydration process [65]. A liquid intake (about 50 mL of natural water) may be not enough to dilute the urine to decrease the electrolytes.

No changes in cTnI, CK-MBm, and CK biomarkers were observed; Type I DCS does not affect cardio-muscular tissues, and oxygen supplementation may play a role in preserving tissue cell integrity.

## 4. Materials and Methods

### 4.1. Subjects

Nine expert technical SCUBA divers (7 males, and 2 females; age 26.8 ± 13.2 years; height 175.2 ± 7.26 cm; weight 76.9 ± 10.1 kg) participated in this study.

All subjects were informed about the aims and provided written informed consent. This study was approved by the Human Ethical Committee (HEC-DSB/04-19) of the Department of Biomedical Science of the University of Padova (Italy) and was carried out in accordance with the standard set by the Declaration of Helsinki [66].

### 4.2. Experimental Protocol

Divers performed a technical dive on the Haven wreck (Arenzano, GE, Italy), and the dive parameters are reported in Table 2. Divers breathed air as the bottom gas and enriched air nitrox 50 (EAN 50, a mixture containing 50% O_2_) and 100% O_2_ during decompression stops.

During the recorded dive, the air temperature ranged from 30 to 33 °C, the wind from 24 to 26 km/h, and humidity from 40 to 62%.

The decompression plan was obtained using the Multideco Software 4.17 (HHS Software Corp, Kingston, ON, Canada) program, and it is reported in Table 3.

### 4.3. Sample Collection

Pre- and post-dive saliva and urine samples were collected. Briefly, for saliva sampling, the subject was instructed on the correct use of a Salivette device (Sarstedt, Nümbrecht, Germany), to refrain from drinking, eating, smoking, brushing their teeth, and to use a mouthwash during the 30 min before salivary collection. Each Salivette was centrifuged (10 min × 3000 g), and approximately 1 mL of saliva was obtained [10]. Urine samples were collected via voluntary voiding in a sterile container provided to the subject. All samples were stored in multiple aliquots at −20 °C [10].

### 4.4. Measurements

#### 4.4.1. ROS Detection by Electron Paramagnetic Resonance

ROS measurements were carried out by an X-band EPR Spectrometer (e-scan, Bruker, Germany). A 1 mM 1-hydroxy-3-methoxycarbonyl-2,2,5,5tetramethyl-pyrrolidine-hydrochloride (CMH, Noxygen Science Transfer & Diagnostics, Germany) spin probe was prepared in buffer solution: Krebs-Hepes buffer (KHB) containing 25 μM deferroxamine methanesulfonate salt (DF) chelating agent and 5 μM sodium diethyldithio-carbamate trihydrate (DETC) at pH 7.4. Saliva and/or plasma was treated with CMH (1:1) and inserted in EPR capillary tube and then placed inside the cavity of EPR spectrometer for acquisition at 37 °C. All spectra were collected using the same acquisition parameters, and the data were converted to absolute concentration values (μmol·min^−1^) using the CP·(3-carboxy-2,2,5,5-tetramethyl-1-pyrrolidinyloxy) stable radical as external reference. Details on the procedures have been previously reported [67,68,69].

#### 4.4.2. 8-Isoprostane (8-iso-PGF2α)

Lipid peroxidation was quantified by immunoassay of 8-isoprostane (8-isoPGF2α) concentration (Cayman Chemical, Ann Arbor, MI, USA, cat N° 516360) in urine as previously described [10,70]. Samples and standard were read in duplicate at a wavelength of 512 nm. Results were normalized by the urine creatinine values. Coefficient of variation (CV) indicated by the manufacturer: inter-assay CV 11.5%; intra-assay CV 8.9%.

#### 4.4.3. Cytokines

Interleukin-6 (IL-6), Interleukin-1β (IL-1β), and tumor necrosis factor (TNF-α) levels were determined by using commercially available enzyme immune assay kits (Cayman Chemical, Ann Arbor, MI, USA, cat N° 501030, cat N° 583311, and cat N° 589201, respectively) following the manufacturer’s instruction, as previously described [27]. The assays are based on a double-antibody sandwich technique. Samples and standard were read in duplicate, signals were spectrophotometrically measured at a wavelength of 450, 410, and 420 nm, respectively. Coefficient of variation (CV) indicated by the manufacturer for IL-6, IL-1β and TNF-α: inter-assay range CV 5.40–26.22%, 2.3–11.4%, and 9.8–40.6%; intra-assay range CV 4.12–9.28%, 1.6–12.7%, and 5.9–111.3%, respectively.

#### 4.4.4. Nitric Oxide Metabolites

Nitric Oxide metabolites ((NOx = (NO_2−_) + (NO_3−_)) level determination was performed by the spectrophotometric method to Griess reagent, in urine, utilizing a commercial colorimetric assay kit (Cayman Chemical, Ann Arbor, MI, USA, cat N° 780001) as previously described [28]. Samples and standard were read in duplicate at a wavelength of 540 nm. Coefficient of variation (CV) indicated by the manufacturer: inter-assay CV 3.4%; intra-assay CV 2.7%.

#### 4.4.5. Creatinine and Neopterin Concentrations

Urinary creatinine and neopterin concentrations were measured using high-pressure liquid chromatography (HPLC), as previously described [58]. Analytic separations were performed at 50 °C on a 5 μm Discovery C18 analytical column (250 × 4.6 mm I.D., Supelco, Sigma-Aldrich) at a 0.9 mL·min^−1^ flow rate. The calibration curves were linear over the ranges of 0.125–1 μmol.L^−1^ and 1.25–10 mmol.L^−1^ for neopterin and creatinine levels. Inter-assay and intra-assay coefficients of variation were <5%.

#### 4.4.6. Uric Acid, and Electrolytes

Urine samples were also collected to determine levels of urea, uric acid, sodium (Na), potassium (K), chlorine (Cl), magnesium (Mg), calcium (Ca), and total protein. They were measured by autosampler of a Roche Cobas^®^ 6000 analyzer (Roche Diagnostics, Basel, Switzerland). The method was previously described [71]. The reported total imprecision was <2.8%, while the intra assay CV% was <1.8%.

#### 4.4.7. Cardio-Muscular Biomarkers

Creatine kinase (C.K.), creatine kinase isoenzyme (CK-MBm), and troponine I (cTnI) were measured only pre- and post-HBOT in subjects with decompression illness, by plasma sample using a Roche Cobas^®^ 6000 analyzer. The method was previously described [72]. The reported total imprecision was <2.8%, while the intra assay CV% was <1.8%.

### 4.5. Scale for Assessment of Physical Fatigue, Pain, and Subjective Perception

The subject-perceived exertion was assessed immediately after diving based on the physical sensations, and muscle fatigue was assessed by the Borg Rate of Perceived Exertion scale (RPE) [73]. Subjective mood, general wellness, happiness/unhappiness, restfulness/tiredness, hot/cold, chills, headaches, nausea, stomachache, calm/agitation, and pain were evaluated using a 1–100 mm visual analog scale (VAS) score. The VAS is a simple, efficient, and reliable method widely employed both in research and clinical practices [74,75,76]. This scoring system was previously suggested for assessing discomfort and general malaise [77].

### 4.6. Description Case of Decompression Sickness, Timing Biological Samples Collection, and Hyperbaric Oxygen Therapy Treatment (HBOT)

Within this experimental session, we reported the case of an expert technical SCUBA diver (age: 36 y; height: 173 cm; weight: 73 kg; BMI: 24.4) in good health and without any past episode of DCS. He does not drink alcoholic beverages. He started diving in 2008, achieving his highest certification (TDI Advanced Trimix) after 12 years. During the week, he sporadically practices swimming.

Similarly to the other volunteers, the injured diver performed the above-described dive on the Haven wreck. Immediately after surfacing, the diver began to feel intense pain at the left elbow without any sign of skin symptoms compatible with Type I DCS. The injured diver was treated with 100% oxygen for 80 min, interrupting the administration every 20 min to drink water.

**Figure 4:**
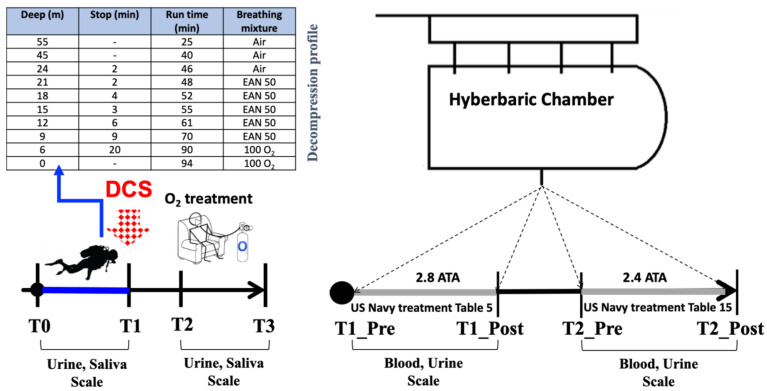
To the **left**, **above**, is the table with the SCUBA decompression profile. The **bottom** is the experimental timeline and timing of HBOT treatment. In detail, saliva and urine samples were collected at the following points: T0: pre-dive; T1: post-dive and simultaneously DCS onset; T2: 3 h post-DCS symptom onset and pre-oxygen breathing; T3: 6 h post-DCS symptom onset and post-oxygen breathing. At the hospital, blood and urine samples were drawn immediately before and after HBOT, respectively, in the first session (T1_pre and T1_post) and second session (T2_post). Scale assessments (BORG and/or VAS) were also performed as indicated in the experimental timeline protocol.

Eight hours after the DCS symptom onset, the diver went to the hyperbaric center of “ASST Ospedale Ca Granda” in Milan. He was treated immediately following the U.S. Navy Treatment Table 5 at 2.8 ATA and the day after with Table 15 at 2.4 ATA (Figure 4). Table 5 treatment consists of a modified compression phase about 9 min long to a depth of 18 msw under 100% oxygen and 2 oxygen cycles lasting 20 min each with short air intervals. Then, the patient is decompressed to about 9 msw, exposed to 2 oxygen cycles lasting 30 min each, and slowly returned to surface pressure; the total elapsed time is about 135 min, excluding the descent. The next day, the diver was treated at 2.4 ATA, with a compression phase about 14 min long to a depth of 14 msw under 100% oxygen and 2 oxygen cycles lasting 30 min each with a short air interval, and slowly returned to surface pressure over the course of 14 min; the total elapsed time is about 93 min, including the descent.

Immediately before and after HBOT, blood and urine samples were drawn. The injured diver participated in a research project on oxidative stress and inflammation status in technical diving, where biological fluids such as urine and saliva were collected as previously reported [10,28,71,78] and described above (see Section 4.3).

Biological samples were collected as follows:

- T0: 30 min before the dive.
- T1: 30 min after surfacing and DCS symptom onset.
- T2: 3 h DCS symptom onset.
- T3: 6 h DCS symptom onset.
- T1_pre: before HBOT treatment at 2.8 ATA (Table 5).
- T1_post: after HBOT treatment at 2.8 ATA (Table 5).
- T2_pre: before HBOT treatment at 2.4 ATA (Table 15).
- T2_post: after HBOT treatment at 2.4 ATA (Table 15).

In the hyperbaric center, venous blood samples (about 5 mL) were collected in EDTA and LH tubes (Vacuette tube, Greiner bio-one, Kremsmünster, Austria) before (Pre) and after (Post) each hyperbaric treatment (n = 2). Blood samples were centrifuged at 3000× *g* at 4 °C (5702R, Eppendorf, Germany) for 10 min to separate plasma. Multiple aliquots were immediately frozen and stored at −80 °C.

**Figure 5:**
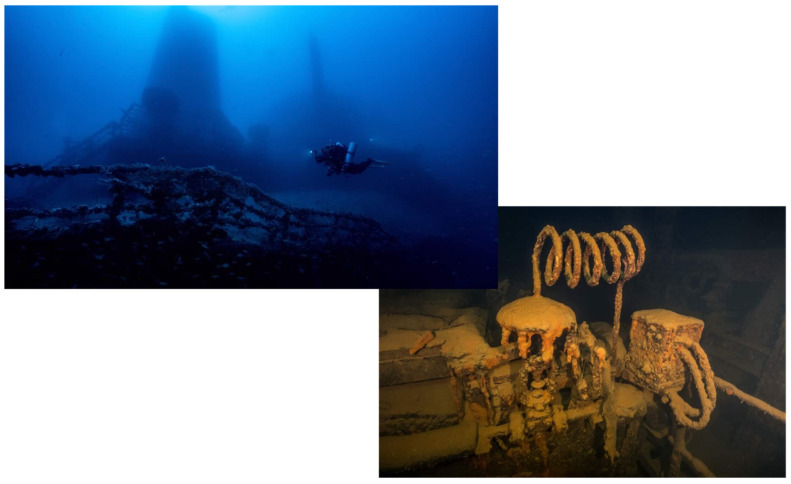
Dive on the Haven oil tanker wreck.

All methods used have been previously described in Section 4.4. Figure 4 shows the experimental protocol and the timeline for biofluid collection. In Figure 5 the Haven oil tanker wreck.

### 4.7. Statistical Analysis

Statistical analyses were performed using the GraphPad Prism software package for Mac (GraphPad Prism 10.3.1, GraphPad Software Inc., San Diego, CA, USA). After the Shapiro–Wilk normality test, statistical analyses were performed using non-parametric tests. The Wilcoxon matched-pairs signed rank test was used, and significant difference was set at *p* < 0.05. In the text, values are expressed as the mean ± standard deviation of the mean (SD). Change Δ% estimation [((post-value − pre-value)/pre-value) ∗ 100)] was also reported in the text.

## 5. Conclusions

The observed oxidative stress and pro-inflammatory biomarkers increase reflected the body’s exposure to high pO_2_ and physical effort during a technical dive. Despite the ROS increase, we observed a NOx augmentation after the dive. In the DCS treatment, HBOT played a significant role in mitigating inflammation and oxidative stress, as observed by the reduction in 8-iso-PGF2α, IL-6, IL-1β, and TNFα. The absence of significant changes in cardio-muscular markers (CK-MB, CK) suggests that Type I DCS did not affect cardiac and muscle tissue.

This observation allowed us to describe the kinetics of oxy-inflammation biomarkers via the resting baseline value to the DCS onset until the HBOT sessions. We reconstructed the temporal progression of the increase in oxidative stress, inflammatory state, and renal damage. In addition to the increase in oxy-inflammation markers, NOx levels increased after the dive, likely as result of the physical exertion, and this trend continued during HBOT. Furthermore, electrolyte levels, especially Mg and urea, decreased as a consequence of dehydration and inflammation, probably associated with DCS. Overall, the data show that HBOT helped counteract the oxidative and inflammatory stress, improving physiological responses in the DCS-injured diver.

## Figures and Tables

**Table 1:** Uric acid, urea, electrolytes (Na, K, Cl, Mg, Ca), and total protein levels at the baseline (T0) and post-immersion (T1) in all divers, and recorded value in the injured subject, 3 h (T2), and 6 h post starting DCS symptoms (T3). On the right side of the table, values recorded pre- and post-HBOT on the first and second day. Statistically significant differences comparisons are displayed as: *, *p* < 0.05; **, *p* < 0.01; and ***, *p* < 0. 001; n.s. not significant.

	All Divers			DCS Subject				HBOT			
	Pre	Post	*p*	T0	T1	T2	T3	1_Pre	1_Post	2_Pre	2_Post
**Uric acid (mg/dL)**	17.9 ± 9.6	14.0 ± 5.7	*	41.5	23.3	32.9	12.2	14.1	27.6	29	31
**Urea (mg/dL)**	1071.1 ± 391.1	777.4 ± 338.5	**	1516	1309	1156	347	302	678	1402	1228
**Na (mmol/L)**	133.5 ± 37.3	136.8 ± 55.9	n.s.	109	100	46	7	13	17	44	57
**K (mmol/L)**	52.3 ± 29.7	53.1 ± 23.2	n.s.	56.6	95.4	66.7	13.6	8.2	22.6	83	60.8
**Cl (mmol/L)**	133.8 ± 34.0	142.6 ± 48.7	n.s.	109	143	72	15	15	15	94	83
**Mg (mg/dL)**	9.9 ± 5.7	5.9 ± 5.5	***	20.7	17.1	8.8	1.2	1.2	1.2	16.9	19.1
**Ca (mg/dL)**	21.4 ± 8.9	16.7 ± 10.3	n.s.	30.3	24.5	11.7	1.7	2.2	2.9	26.9	31.3
**Total protein** **(mg/dL)**	50	50	n.s.	50	50	50	152	153	100	50	50

**Table 2:** Diving parameters.

Dive Parameters	
Maximum depth (m)	55 m
Bottom time (min)	40
Run time (min)	94
Computer algorithm	7HL16-B
Gradient factor (GF)	35/85
Bottom gas	Air
Decompression gas	EAN 50 + 100% O_2_
Water temperature (surface)	27 °C
Water temperature (bottom)	15 °C

**Table 3:** Decompression plan of the dive.

Depth (m)	Stop (min)	Run Time (min)	Breathing Mixture
55	-	25	Air
45	-	40	Air
24	2	46	Air
21	2	48	EAN 50
18	4	52	EAN 50
15	3	55	EAN 50
12	6	61	EAN 50
9	9	70	EAN 50
6	20	90	100% O_2_
0	-	94	100% O_2_

## Data Availability

The datasets used and analyzed during the current study are available from the corresponding author upon reasonable request.
